# Effects of dietary supplementation with bacteriocin-like inhibitory substance-producing *Weissella cibaria* XN-2a on growth performance, immune responses, and gut microbiota in crucian carp (*Carassius auratus*)

**DOI:** 10.3389/fmicb.2026.1953174

**Published:** 2026-09-14

**Authors:** Nuo Xu, Tianshuo Guo, Zhuolei Yang, Yuhang Zheng, Yu Wang, Wanli Sha, Baishuang Yin, Wenlong Dong

**Affiliations:** 1 College of Animal Science and Technology, JiLin Agricultural Science and Technology College, Jilin, China; 2 Jilin Provincial Key Laboratory of Preventive Veterinary Medicine, Jilin, China; 3 Jilin Province Technology Innovation Center of Pig Ecological Breeding and Disease Prevention and Control, Jilin, China; 4 Jilin Province Cross Regional Cooperation Technology Innovation Center of Porcine Main Disease Prevention and Control, Jilin, China

**Keywords:** bacteriocin-like inhibitory substance, *carassius auratus*, immunomodulation, metagenomics, probiotic

## Abstract

**Introduction:**

The intensive farming of crucian carp (*Carassius auratus*) often compromises fish health and increases disease susceptibility, driving interest in probiotic interventions as sustainable alternatives to antibiotics. However, the *in vivo* efficacy and mechanistic basis of *Weissella cibaria* strains in freshwater fish remain poorly understood.

**Methods:**

A bacteriocin-producing strain, *Weissella cibaria* XN-2a, was isolated from fish viscera and supplemented in the diet of crucian carp at an initial viable dose of 2.74 × 10^10^ CFU/g feed (freshly prepared and replaced every 3 days) for 30 days, followed by a challenge with *Aeromonas hydrophila*. Growth performance, immune parameters, intestinal histology, and gut metagenomic profiles were compared between the probiotic-fed group (XS) and the control group (XD).

**Results:**

Probiotic supplementation significantly improved weight gain (28.17% vs. 13.98%), feed conversion ratio (1.88 ± 0.32 vs. 2.58 ± 0.27, *p* < 0.05), and post-challenge survival (50% vs. 0%) (*p* < 0.001). The XS group exhibited enhanced intestinal morphology (higher mucosal fold height and FH/IFD ratio, lower inter-fold distance), elevated IgM and SOD activities, and reduced post-challenge IL-6 and MDA levels. Metagenomic analysis revealed that the probiotic reshaped the gut microbiota from a pathogen-associated virulence profile (LPS, flagella, enterobactin) toward a commensal profile enriched in T6SS/T2SS and alternative siderophores, without increasing the overall antibiotic resistance burden. The butanoate metabolism pathway showed differences consistent with increased butyrate synthesis potential, with enrichment of butyrate-synthetic enzymes (e.g., enoyl-CoA hydratase, acetolactate decarboxylase) in XS and butyrate-diverting enzymes in XD. CAZy profiling further showed enrichment of antibacterial (GH25/GH73) and antioxidant (AA4) gene families in XS, with *Weissella* as a major contributor.

**Discussion:**

These findings suggest that dietary *Weissella cibaria* XN-2a enhances growth performance, intestinal health, and disease resistance in crucian carp, potentially through a dual mechanism involving bacteriocin-mediated microbiota modulation and enhanced butyrate synthesis potential. Further investigations, including direct metabolite quantification, are warranted to validate these mechanistic inferences.

## 1 Introduction

The global aquaculture industry has experienced remarkable expansion over recent decades, becoming a critical source of animal protein to meet the demands of a growing population (Zaki et al., 2020). To maximize yield and economic efficiency, intensive farming practices with high stocking densities have become widespread. However, these conditions often induce chronic stress in cultured fish, compromising physiological homeostasis and manifesting as immunosuppression, suppressed growth, impaired reproduction, and deteriorated flesh quality—collectively heightening susceptibility to disease outbreaks and causing significant economic losses (Ran et al., 2016; Mahmoud et al., 2021).

Among the species affected by these challenges, the *Carassius auratus* (*C. auratus*) is a species of substantial economic importance in Chinese freshwater aquaculture. In the aquaculture context, this species is commonly referred to as crucian carp, distinguishing it from the ornamental goldfish varieties of the same species. Renowned for its strong environmental resilience, rapid growth rate, desirable palatability, and high nutritional value, it has become a staple species widely cultivated across the country (Du et al., 2021; Shi et al., 2025). Its significant commercial status is underscored by an annual production of 2,850,316 tons in 2025, ranking first among all freshwater farmed fish species in China Bureau of Fisheries of the Ministry of Agriculture and Rural Affairs of China National Aquatic Technology Extension General Station China Society of Fisheries, 2026. Globally, the broader category of “Carps, barbels and other cyprinids,” which includes crucian carp, reached approximately 3.236 million tons in aquaculture production in 2023, further highlighting the substantial contribution of crucian carp to global aquaculture (FAO, 2025; Cui et al., 2026). Ensuring the health and productivity of crucian carp under intensive farming is therefore essential for the sustainable development of the sector. Beyond its aquacultural value, *C. auratus* also serves as a well-established experimental model in fish biology. Its moderate body size, ease of handling, and well-characterized physiology and immunology make it particularly suitable for nutritional and immunological studies (Shi et al., 2025; Cui et al., 2026). In addition, the ornamental variant of this species is widely known as the goldfish in the global aquarium trade. However, the present study focuses specifically on the aquaculture form, given its greater relevance to food production and the need for sustainable health management strategies in intensive farming systems. These attributes collectively make crucian carp an ideal subject for evaluating the effects of probiotic supplementation on growth, immunity, and intestinal health in a commercially important fish species.

Traditionally, antibiotics were used for disease control in aquaculture, but this practice is now recognized as unsustainable due to the emergence of antibiotic resistance and environmental residues (Cabello, 2006; Hektoen et al., 1995; Abdel-Latif et al., 2023). This has driven the search for alternative strategies, among which probiotics have emerged as a promising approach. Probiotics are live microbial supplements that confer health benefits by improving the host's intestinal microbial balance. Among the diverse beneficial effects of probiotics, the production of antimicrobial proteins and peptides—such as bacteriocins—is of particular interest, as these compounds can directly inhibit pathogenic bacteria through specific mechanisms (Ghosh, 2025; Hoseinifar et al., 2021). Among lactic acid bacteria (LAB), the genus *Weissella* has attracted attention for its probiotic attributes, including the production of antimicrobial compounds (e.g., bacteriocins) and immunomodulatory properties (Contente et al., 2021). However, *in vivo* studies on *Weissella cibaria* (*W. cibaria*) in aquaculture, particularly in economically important freshwater fish like crucian carp, remain limited.

To bridge this critical knowledge gap, the present study isolated a BLIS-producing strain, *W. cibaria* XN-2a, from the intestinal tract of crucian carp obtained from fish viscera collected as processing waste at a seafood market and incorporated it into feed. Through a controlled 30-day feeding trial with crucian carp, this research systematically evaluated the effects of dietary *W. cibaria* XN-2a supplementation on (1) key growth performance parameters, (2) immunomodulatory capacity and disease resistance against the common pathogen *Aeromonas hydrophila* (*A. hydrophila*), and (3) modulation of the host intestinal microbiota composition. It should be noted that while metagenomic and phenotypic analyses can reveal strong associations, they do not establish causality. Therefore, the mechanistic interpretations presented herein are hypothesis-generating and intended to guide future functional studies. Nevertheless, the observed associations—including improved growth performance, enhanced immune parameters, and better intestinal health — hold direct practical implications for the crucian carp aquaculture industry. These findings suggest that *W. cibaria* XN-2a may offer a concrete, natural alternative to antibiotic growth promoters. If validated in future studies, this strain could support the development of a specialized probiotic feed additive, promoting healthier fish stocks, reducing disease-related losses, and enhancing the overall productivity and environmental sustainability of crucian carp farming.

## 2 Materials and methods

### 2.1 Bacterial isolation, genomic DNA extraction and 16S rRNA gene sequencing

Visceral samples of crucian carp were collected as processing waste from a seafood market in Changchun, China, and transported to the laboratory at 4 °C. The intestinal tract was then dissected and used for bacterial isolation and purification. For bacterial isolation, the samples were serially diluted with sterile physiological saline (0.85% NaCl), spread onto de Man, Rogosa and Sharpe (MRS) agar (Hopobio, China), and incubated at 37 °C for 24 h. Distinct colonies were purified by successive streaking, and presumptive lactic acid bacteria were identified by Gram staining. Among the isolates, strain XN-2a showed the strongest antagonistic activity against *A. hydrophila* ATCC 7966 and was selected for further study. The strain was preserved at −80 °C in MRS broth supplemented with 20% (v/v) glycerol.

Genomic DNA was extracted from pure cultures of XN-2a using a bacterial genomic DNA rapid extraction kit (Aidlab Biotechnologies, China) according to the manufacturer's protocol for Gram-positive bacteria. DNA concentration and purity (A_2_60/A_2_80) were measured with a NanoDrop One spectrophotometer (Thermo Fisher Scientific, USA).

The 16S rRNA gene was amplified using universal primers 27F and 1492R under standard PCR conditions (initial denaturation at 94 °C for 5 min; 30 cycles of 95 °C for 1 min, 55 °C for 1 min, 72 °C for 1 min; final extension at 72 °C for 10 min). Amplicons of approximately 1,500 bp were verified by agarose gel electrophoresis, purified, and subjected to bidirectional Sanger sequencing at General Biology (Anhui, China). The resulting forward and reverse sequences were assembled and manually curated to obtain a high-quality consensus sequence for taxonomic identification.

### 2.2 Whole genome sequencing of XN-2a

Strain XN-2a was cultured in MRS broth at 37 °C for 24 h. The cell pellet was collected by centrifugation (3,000 × g, 15 min) and shipped on dry ice to Shanghai Majorbio Bio-Pharm Technology Co., Ltd. for sequencing. DNA quality was assessed by measuring purity (A_2_60/A_2_80 and A_2_60/A_2_30 ratios) and integrity (agarose gel electrophoresis), and strain identity was confirmed by 16S rRNA gene Sanger sequencing.

A sequencing library was prepared using the NEXTFLEX Rapid DNA-Seq Kit (PerkinElmer, USA) after fragmentation to −400 bp. Paired-end sequencing (2 × 150 bp) was performed on an Illumina NovaSeq 6000 platform. Raw reads were quality-filtered with fastp v0.23.0, and the clean reads were assembled into a complete genome using Unicycler v0.4.8 (Wick et al., 2017).

Coding sequences (CDSs) were predicted with GeneMarkS v4.3 (Besemer and Borodovsky, 2005). Plasmids were identified using PlasFlow v1.1. Biosynthetic gene clusters were annotated with antiSMASH v7.0.0. Antibiotic resistance genes (ARGs) were predicted by aligning proteins against the Comprehensive Antibiotic Resistance Database (CARD v4.0.1) using the Resistance Gene Identifier (RGI v6.0.5). Virulence factors were identified by alignment against the Virulence Factors Database (VFDB, version 20240301).

### 2.3 Acid and bile salt tolerance assays

The tolerance of *W. cibaria* XN-2a to simulated gastric acid and intestinal bile salts was evaluated *in vitro* (Turpin et al., 2011; Anglenius et al., 2023). The strain was pre-cultured in MRS broth at 37 °C under anaerobic conditions to late exponential phase (OD_600_ ≈ 1.0). Cells were harvested by centrifugation (8,000 × g, 5 min, 4 °C), washed twice with sterile physiological saline (0.85% NaCl), and resuspended to ≈1 × 10^8^ CFU/mL.

For acid tolerance, MRS broth was adjusted to pH 2.0 (simulated gastric fluid) or kept at native pH 6.5 (control). Aliquots (9 mL) were inoculated with 1 mL of cell suspension and incubated statically at 37 °C. Viable counts were determined at 0, 2, 4, 6 and 8 h by serial dilution and plating on MRS agar.

For bile salt tolerance, MRS broth was supplemented with 0.3% (w/v) oxgall (simulated intestinal fluid) or left unsupplemented (control). Inoculation was performed as above, and cultures were incubated at 37 °C with shaking at 120 rpm. Viable counts were determined at 0, 2, 4, 6 and 8 h.

All assays were performed in triplicate. $Survival\;rate\left(\%\right)\;=\frac{N_{test}\left(T\right)}{N_{control}\left(T\right)}\times 100$.

Where *N*_*test*_(*T*) and *N*_*control*_(*T*) represent the viable counts (CFU/mL) in the probiotic-treated and control groups at each sampling time point, respectively. All counts were performed in triplicate.

### 2.4 Safety assessment: hemolytic activity assay

The hemolytic activity of *W. cibaria* XN-2a was assessed using a blood agar plate method. Briefly, *W. cibaria* XN-2a and *A. hydrophila* (as a positive control) were cultured to the mid-logarithmic phase in MRS and BHI broth, respectively. Subsequently, 2 μL of each bacterial suspension was streaked onto Blood Agar plates (BKMAMLAB, containing 5% defibrinated sheep blood). The inoculated plates were incubated aerobically at 37 °C for 18 h. Following incubation, the plates were examined for the presence of clear zones (β-hemolysis) around the bacterial streaks (Yin et al., 2023). The hemolytic activity of *W. cibaria* XN-2a was evaluated qualitatively by direct visual observation and compared to that of the positive control, *A. hydrophila*.

### 2.5 Antimicrobial activity measurement

Strain XN-2a was cultivated in 300 mL of MRS broth (Hopobio, China) at 37 °C for 48 h. Bacterial cells were removed by centrifugation at 3000 × g for 15 min at 4 °C using a TGL16M centrifuge (Yancheng Kate Experimental Instrument Co., Ltd., China). The supernatant was neutralized to pH 6.5 with 1 mol/L NaOH to eliminate organic acid effects, then treated with catalase (1 mg/mL, 37 °C, 2 h) to degrade residual hydrogen peroxide, followed by heat inactivation at 100 °C for 5 min. All samples were filtered through a 0.22 μm polyethersulfone (PES) membrane filter (13 mm diameter; Tianjin Keyilong Experimental Equipment Co., Ltd., China) to obtain cell-free supernatant (CFS) (Simova et al., 2009).

Antibacterial activity was assessed using the Oxford cup method (Wu et al., 2025). Briefly, 50 μL of an activated *A. hydrophila* ATCC 7966 suspension was spread onto Mueller-Hinton (MH) agar plates. Sterile Oxford cups (8 mm) were placed on the agar, and 100 μL of CFS was added into each cup. After standing at room temperature for 1 h to allow diffusion, plates were incubated at 37 °C for 12 h. The inhibition zone diameter around each cup was measured using a vernier caliper (accuracy 0.02 mm).

### 2.6 Growth kinetics and antibacterial activity profiling

Growth kinetics and antimicrobial production were evaluated following a previously described method. XN-2a was inoculated at 2% (v/v) into MRS broth and cultured at 37 °C for 36 h. To monitor growth, OD_600_ was measured at 2-h intervals. For antibacterial activity, culture samples at selected time points were centrifuged at 7,378 × g for 5 min at 4 °C using a laboratory centrifuge (Sigma, Germany). The supernatant was sterile-filtered (0.22 μm) to obtain CFS, and the antibacterial activity against *A. hydrophila* ATCC 7966 was determined using the standard Oxford cup assay on MH agar plates.

### 2.7 Production, concentration and characterization of concentrated crude BLIS

Crude BLIS (bacteriocin-like inhibitory substance) was extracted following Zheng et al. (2025) (Zheng et al., 2025). XN-2a was cultured in MRS broth at 37 °C for 24 h, with this time point selected based on the growth kinetics and antibacterial activity profiling described above (Section 2.6). CFS was prepared as described in section 2.5, then concentrated ten-fold using a rotary evaporator at 50 °C and filtered through a 0.22 μm membrane. The concentrate (crude BLIS) was stored at 4 °C for subsequent characterization

Stability tests were performed on crude BLIS under various physicochemical conditions using the agar-well diffusion assay described in section 2.5 (in triplicate).

#### 2.7.1 Effect of temperature

Aliquots of the crude BLIS extract were incubated at different temperatures (40 °C, 60 °C, 80 °C, 100 °C) for 1 h. After cooling to room temperature, the residual activity was compared to that of an untreated aliquot stored at 4 °C (control).

#### 2.7.2 Effect of pH

The pH of crude BLIS was adjusted to values ranging from 3.0 to 10.0 using sterile 1 mol/L HCl or NaOH. After incubation at 37 °C for 2 h, the pH was readjusted to the original value. Residual activity was compared to a control incubated without pH adjustment.

#### 2.7.3 Sensitivity to proteolytic enzymes

Crude BLIS was treated with proteinase K, trypsin, pepsin, or papain at a final concentration of 1 mg/mL. The pH of each reaction was adjusted to the optimal range for the respective enzyme. After incubation at 37 °C for 2 h, enzymes were inactivated by heating at 100 °C for 10 min. A control sample was processed without enzyme. Residual antibacterial activity was then assessed.

### 2.8 Scanning electron microscope (SEM)

The effect of BLIS from *W. cibaria* XN-2a on the membrane integrity of *A. hydrophila* ATCC 7966 was examined by SEM. Bacterial cells (1 × 10^8^ CFU/mL in PBS) were treated with BLIS for 2 h at 37 °C, while untreated cells served as control. Samples were fixed, dehydrated, critical-point dried (Quorum K850 dryer), sputter-coated with gold (Hitachi MC1000 coater), and observed under a Hitachi SU8100 SEM at 5.0 kV (Polat et al., 2024).

### 2.9 Cell viability assay (live/dead staining)

Membrane damage was assessed using SYTO-9/PI staining (Servicebio Bacterial/Yeast Viability Kit). Bacterial cells (1 × 10^8^ CFU/mL in PBS) were treated with BLIS for 2 h at 37 °C, while untreated cells served as control. After washing, samples were stained with SYTO-9/PI solution (1 μL dye mix per 100 μL suspension) for 15 min in the dark, then examined under a fluorescence microscope (Nikon Eclipse Ti-E) using FITC and Texas Red channels.

### 2.10 Fish management

Healthy crucian carp with an average body weight of 8.78 ± 0.22 g (mean ± SD) were obtained for this study. The experiment was conducted at the College of Animal Science and Technology, JiLin Agricultural Science and Technology College, over a 45-day period. Following a 15-day acclimation period, fish were randomly assigned to two groups: an experimental group and a control group, with three replicate tanks per group (30 fish per tank). Fish were maintained in aquaria, each containing 70 L of dechlorinated tap water. To ensure continuous aeration, each tank was equipped with an air stone connected to a central air compressor system. Fecal matter was removed daily, and approximately 30% of the water volume was exchanged to maintain water quality. The fish were kept under a controlled photoperiod of 12 h light: 12 h dark throughout the experimental period (Mahmoud et al., 2021). The 30-day feeding period was selected based on the high metabolic rate and rapid intestinal epithelial turnover of juvenile fish, which typically enables detection of probiotic-induced physiological changes within 2–4 weeks. This duration aligns with standard probiotic feeding trials in cyprinid fish and was designed to capture the early critical window for intestinal colonization, immune modulation, and growth responses prior to the *A. hydrophila* challenge (Qi et al., 2025; Jiang et al., 2020).

### 2.11 Preparation, stability assessment, and feeding of experimental diets

The experimental diets were prepared as follows. The basal diet used throughout the study was a commercial extruded fish feed (Model 152, Tongwei Agricultural Development Co., Ltd.). Its proximate composition and ingredient list are presented in Table 1. For the control group (CON), fish were fed the basal diet without any modification. For the probiotic group (XN-2a), *W. cibaria* XN-2a was cultured in MRS broth at 37 °C for 24 h. Bacterial cells were harvested by centrifugation (3,000 × g, 15 min, 4 °C), washed, and resuspended in sterile saline (0.85% NaCl) to form a dense cell paste. The target dose of approximately 10^10^ CFU/g feed was selected based on a previous dose-response study in *C. auratus* (Vadassery and Pillai, 2020). This paste was then evenly sprayed onto the basal diet pellets under constant manual mixing. Sodium carboxymethyl cellulose (CMC-Na, 0.3% of diet weight) dissolved in sterile water was subsequently added as a binder to enhance bacterial adhesion (Mohammady et al., 2025). The coated feed was dried in a forced-air drying oven at 28 °C for 4–6 h until reaching the original dry and loose state (Zhang et al., 2024).

**Table 1:** Nutrient levels of the basal diet (152-type floating feed for fish).

Nutrient	Content (%)
Crude protein	≥32.0
Crude fat	≥5.0
Crude fiber	≤ 12.0
Ash	≤ 15.0
Calcium	0.5–2.0
Total phosphorus	≥1.0
Moisture	≤ 11.0
Lysine	≥1.6

The diet contained fish meal, soybean meal, rapeseed meal, cottonseed meal, wheat flour, L-lysine, DL-methionine, vitamin premix, mineral premix, and other ingredients. Values are manufacturer-guaranteed ranges. The feed is an extruded diet (Model 152, Tongwei Agricultural Development Co., Ltd., diameter 2.0 mm). Both groups received the same basal diet batch; the probiotic diet was coated with the bacterial suspension and dried at 28 °C. The viable cell count in the coated feed was verified by a pre-experimental stability test (Section 2.11).

### 2.12 Storage stability assessment of the probiotic feed (pre-experiment)

A pre-experimental stability assessment was conducted to determine the viable cell count dynamics in the coated feed. Immediately after preparation, the feed was stored at 4 °C in sealed sterile bags. Samples (approx. 1.0 g) were aseptically collected in triplicate at day 0 (post-drying), day 3, and day 7 of storage. Each sample was homogenized with 9 mL of sterile saline in a sterile mortar by grinding for 5 min. After brief sedimentation, the supernatant was serially diluted (10-fold), plated onto MRS agar, and incubated anaerobically at 37 °C for 48 h. Colonies were counted, and viable bacteria were expressed as colony-forming units per gram of feed (CFU/g).

Quantitative analysis revealed that the viable count of the freshly prepared feed (after drying and within 3 days of storage at 4 °C) was 2.74 × 10^10^ CFU/g. This count decreased by approximately 89% to 3.00 × 10^9^ CFU/g after 7 days of storage.

Based on the results of this stability assessment, which indicated a significant decline in viable counts by day 7, a feeding protocol was established to ensure consistent probiotic dosage. Therefore, freshly prepared *W. cibaria*-coated feed was supplied to the XN-2a group every 3 days throughout the feeding trial. The control group received the uncoated basal diet on the same schedule. During the feeding trial, each tank received 3 g of feed per feeding, which was completely consumed within 5 min, minimizing nutrient leaching and bacterial loss in the water.

### 2.13 Sample collection

For subsequent analysis, fish were fasted for 24 h prior to sampling for blood and intestinal tissue collection. This fasting period minimized the influence of recent feeding on blood biochemical and immune parameters and standardized the physiological state across all groups, following standard practices in fish nutritional studies (Qiu et al., 2025; Cai et al., 2025). Fish used for intestinal content collection (Section 2.12.1) were not fasted, as fasting would compromise the recovery of sufficient microbial DNA for metagenomic sequencing (Wu et al., 2015; Gajardo et al., 2016). At the time of sampling, fish were anesthetized by immersion in a buffered solution of tricaine methanesulfonate (MS-222, CAS No. 886-86-2, purity 98%, Shanghai Aladdin Biochemical Technology Co., Ltd., China) at 100 mg/L, with an equal weight of sodium bicarbonate added to maintain neutral pH (Elshafey et al., 2026). Anesthesia was achieved within approximately 2–4 min, confirmed by complete loss of equilibrium and absence of response to tail-pinching. Blood and tissue samples were then collected while the fish remained under deep anesthesia (opercular arrest). After sampling, all fish were euthanized by spinal cord severance; MS-222 immersion alone is not reliably lethal to crucian carp, so physical cerebral disruption was added as a secondary step (Balko et al., 2018). If any signs of recovery appeared during the procedure, fish were immediately returned to the 100 mg/L MS-222 solution until deep anesthesia was re-established, after which sampling was continued and spinal cord severance was performed.

#### 2.13.1 Intestinal content collection

From each group, 20 fish were randomly selected and dissected aseptically under anesthesia. Because the intestinal content from a single juvenile fish was insufficient for metagenomic sequencing, contents from two fish were pooled to form one biological replicate, yielding ten replicates per group (*n* = 10). Intestinal contents were collected from the posterior intestine following a standardized protocol (Wu et al., 2015; Gajardo et al., 2016). Briefly, contents from two fish were pooled to form one biological replicate, yielding ten replicates per group. The pooled samples were immediately flash-frozen in liquid nitrogen and stored at −80 °C for microbial analysis.

#### 2.13.2 Blood plasma collection

Subsequent to the intestinal sampling, blood samples were collected from 30 fish per group. Blood was drawn from the caudal vein using heparinized syringes and transferred into sterile microcentrifuge tubes (Mahmoud et al., 2021). Samples were centrifuged at 4,000 rpm for 15 min at 4 °C to separate serum. Serum samples from individual fish within the same experimental replicate tank were pooled, aliquoted, and stored at −20 °C until analysis of immune parameters. All assays were performed in triplicate.

#### 2.13.3 Intestinal tissue fixation

For histological examination, eight fish per group were dissected under anesthesia. The entire intestine was carefully removed, rinsed with ice-cold physiological saline to remove residual contents, and fixed in 4% paraformaldehyde (PFA) solution at 4 °C for 24 h. Fixed tissues were then processed for paraffin embedding, sectioning, and staining.

### 2.14 Growth performance evaluation in crucian carp

Fish in each tank were counted and bulk-weighed on a weekly basis throughout the 30-day feeding trial to assess growth performance. Key parameters, including weight gain (WG), specific growth rate (SGR), feed conversion ratio (FCR), and survival rate (SR), were calculated using the following formulas:


$$\begin{array}{ccc} WG\;\left(\%\right) & = & \frac{\left(W_{t}-W_{0}\right)}{W_{0}}\times 100 \end{array}$$

$$\begin{array}{ccc} SGR\;\left(\%/day\right) & = & \frac{\left(\ln\;W_{t}-\;ln\;W_{0}\right)}{t}\times 100\\ FCR & = & \frac{Total\;feed\;intake\left(g\right)}{Weight\;gain\left(g\right)}\\ SR\;\left(\%\right) & = & \frac{Final\;number\;of\;fish}{Initial\;number\;of\;fish}\times 100 \end{array}$$

Where W_0_ and W_t_ represent the initial and final average body weight (g) of fish, respectively, and t is the experimental period in days.

### 2.15 Analysis of immunological parameters

The concentrations of trypsin, tumor necrosis factor-alpha (TNF-α), interleukin-6 (IL-6), interleukin-10 (IL-10), immunoglobulin M (IgM), Superoxide dismutase (SOD) and malondialdehyde (MDA) in plasma samples were quantified using their respective commercial enzyme-linked immunosorbent assay (ELISA) kits in strict accordance with the manufacturer's protocols (Nanjing Jiancheng Bioengineering Institute, Nanjing, Jiangsu, China). All assays were performed in triplicate.

### 2.16 Intestinal histopathological analysis (H&E staining)

At the conclusion of the feeding trial, distal intestinal segments from 8 fish per group were collected. Tissues were fixed in 4 % paraformaldehyde, embedded in paraffin, sectioned at 5 μm thickness, and stained with hematoxylin and eosin (H&E) according to standard protocols. The 5 μm thickness was selected as it is the standard and most widely used thickness for routine histological assessment of fish intestinal tissues with H&E staining, providing optimal cellular resolution while maintaining tissue integrity for morphometric measurements. This thickness has been extensively employed in intestinal histopathological studies of crucian carp (Jiang et al., 2020; Wu et al., 2021).

Stained sections were examined under a light microscope (Nikon Eclipse Ci-L, Japan). For each sample, mucosal fold height (FH), mucosal fold width (FW), inter-fold distance (IFD), and mucosal thickness (MT) were measured from six intact, well-oriented mucosal folds, and the average of each parameter was calculated as one data point per fish (*n* = 8 per group). The fold height to inter-fold distance ratio (FH/IFD) was then calculated.

### 2.17 Gut microbiota metagenomic sequencing

Total microbial DNA was extracted from approximately 200 mg of intestinal content using the E.Z.N.A.^®^ Soil DNA Kit (Omega Bio-tek, USA). DNA quality was verified by 1% agarose gel electrophoresis and spectrophotometry. Sequencing libraries were prepared using the NEXTFLEX Rapid DNA-Seq Kit (Bioo Scientific, USA) following fragmentation to ~350 bp. Paired-end sequencing (2 × 150 bp) was performed on an Illumina NovaSeq platform at Majorbio Bio-Pharm Technology Co., Ltd. (Shanghai, China).

Raw reads were quality-filtered and trimmed using Fastp (v0.23.2) to remove adapters and low-quality bases (Chen et al., 2018). Potential host (fish) DNA was removed by alignment to the *C. auratus* genome using BWA (v0.7.17) (Li and Durbin, 2009). High-quality, host-free reads from all samples were co-assembled into contigs using MEGAHIT (v1.2.9) (Li et al., 2015). Open reading frames (ORFs) were predicted from contigs (≥300 bp) using Prodigal (v2.6.3) (Hyatt et al., 2010). CD-HIT (v4.8.1) to construct a non-redundant gene catalog (NRGC) (Fu et al., 2012). Gene abundance in each sample was quantified by mapping reads back to the NRGC with SOAPaligner (v2.21) (Li et al., 2008).

The NRGC was functionally and taxonomically annotated using Diamond (v2.0.15; *e*-value ≤ 1e-5) against the following databases: NCBI NR (taxonomy), KEGG, CARD (antibiotic resistance), and VFDB (virulence factors) (Buchfink et al., 2015).

Raw sequence data have been deposited in the NCBI SRA; accession numbers are listed in Table 2.

**Table 2:** Sequencing data types and accession numbers used in this study.

Data type	Sample name	Accession number
16S rRNA	XN-2a	PX884722
Whole genome sequencing	XN-2a	SRR36343504
Gut microbiota	XS-1	SRR37043784
	XS-3	SRR37043783
	XS-4	SRR37043782
	XS-5	SRR37043781
	XS-6	SRR37043780
	XS-7	SRR37043779
	XS-9	SRR37043778
	XS-10	SRR37043777
	XS-11	SRR37043776
	XS-12	SRR37043775
	XD-3	SRR37043786
	XD-4	SRR37043785
	XD-5	SRR37043774
	XD-7	SRR37043773
	XD-8	SRR37043772
	XD-9	SRR37043771
	XD-11	SRR37043770
	XD-12	SRR37043769
	XD-13	SRR37043768
	XD-14	SRR37043767

### 2.18 Statistical analysis

All data were expressed as the mean ± standard deviation (SD). Comparisons between the experimental and control groups were performed using independent samples *t*-tests (for growth parameters, immune indices, trypsin activity, and antioxidant markers). For IL-6 and IL-10 measured before and after challenge, and for intestinal morphology parameters (FW, MT, FH, IFD, and FH/IFD), two-way repeated measures ANOVA was used with Group and Challenge as the two factors, followed by Bonferroni *post-hoc* tests. Survival after *A. hydrophila* challenge was analyzed using the Kaplan-Meier method, and differences between groups were evaluated by the log-rank (Mantel-Cox) test. Survival curves are presented with 95% confidence intervals. Alpha diversity (Shannon index) was compared using the Wilcoxon rank-sum test, and beta diversity was analyzed by ANOSIM based on Bray-Curtis distances. LEfSe analysis (linear discriminant analysis effect size) was applied to identify differentially abundant ARGs, VFs, KEGG pathways and CAZy families, with an LDA score threshold >2 and *p* < 0.05. For metagenomic enzyme abundances in the butanoate pathway, the Wilcoxon rank-sum test was used. All statistical tests were two-tailed, and significance was set at *p* < 0.05. Visualizations were generated using Origin 2025.

### 2.19 Infection challenges

At the end of the 30-day feeding trial, a bacterial challenge experiment was conducted using *A. hydrophila* ATCC 7966. Prior to the formal challenge, a preliminary LD_50_ assay was performed to determine the appropriate challenge dose. The LD_50_ was determined to be approximately 1.5 × 10^7^ CFU/fish. Fish from each dietary group (30 fish per group, divided into 3 replicate tanks of 10 fish each) were intraperitoneally injected with 0.1 mL of bacterial suspension at a concentration of 1.5 × 10^8^ CFU/mL (verified by plate counting immediately prior to injection), corresponding to 1.5 × 10^7^ CFU per fish—the LD_50_ dose determined in the preliminary assay. A sham-injected control group (30 fish, 3 replicate tanks of 10 fish each) received an equal volume of sterile PBS instead of the bacterial suspension. Mortality was recorded daily for 7 days post-challenge. The relative level of protection (RLP) was calculated according to the formula described by Ruangpan et al. (1986) (Ruangpan et al., 1986):


$$\begin{array}{c} \text{RLP }(\text{\%})\;=\;100\;-(\text{Percent mortality in the treated group}/\\ \;\text{Percent mortality in the control group})\;\times \;100 \end{array}$$

## 3 Results

### 3.1 Genomic and phenotypic characterization of *W. cibaria* XN-2a

Strain XN-2a, isolated from the intestinal tract of crucian carp, was identified as *W. cibaria* by 16S rRNA gene sequencing (>99% identity with reference sequences). The 16S rRNA gene sequence has been deposited in GenBank under accession number PX884722. Average nucleotide identity (ANI) analysis against six *W. cibaria* reference strains ranged from 96.56% to 99.97%, all exceeding the 95% species delineation threshold, while values against *W. confusa* strains were below 80%, confirming taxonomic assignment (Figure 1A) (Figueras et al., 2014). Whole-genome sequencing revealed a single circular chromosome of 2,568,612 bp (GC 44.91%) and two native plasmids. A 41-kb biosynthetic gene cluster (BGC) predicted to encode a type III polyketide synthase (T3PKS) was identified, comprising 31 genes involved in metabolism, membrane transport, translation, and cell wall remodeling, which is consistent with the genetic basis for antimicrobial compound production and supports the strain's probiotic and BLIS-producing potential (Figure 1B).

**Figure 1:**
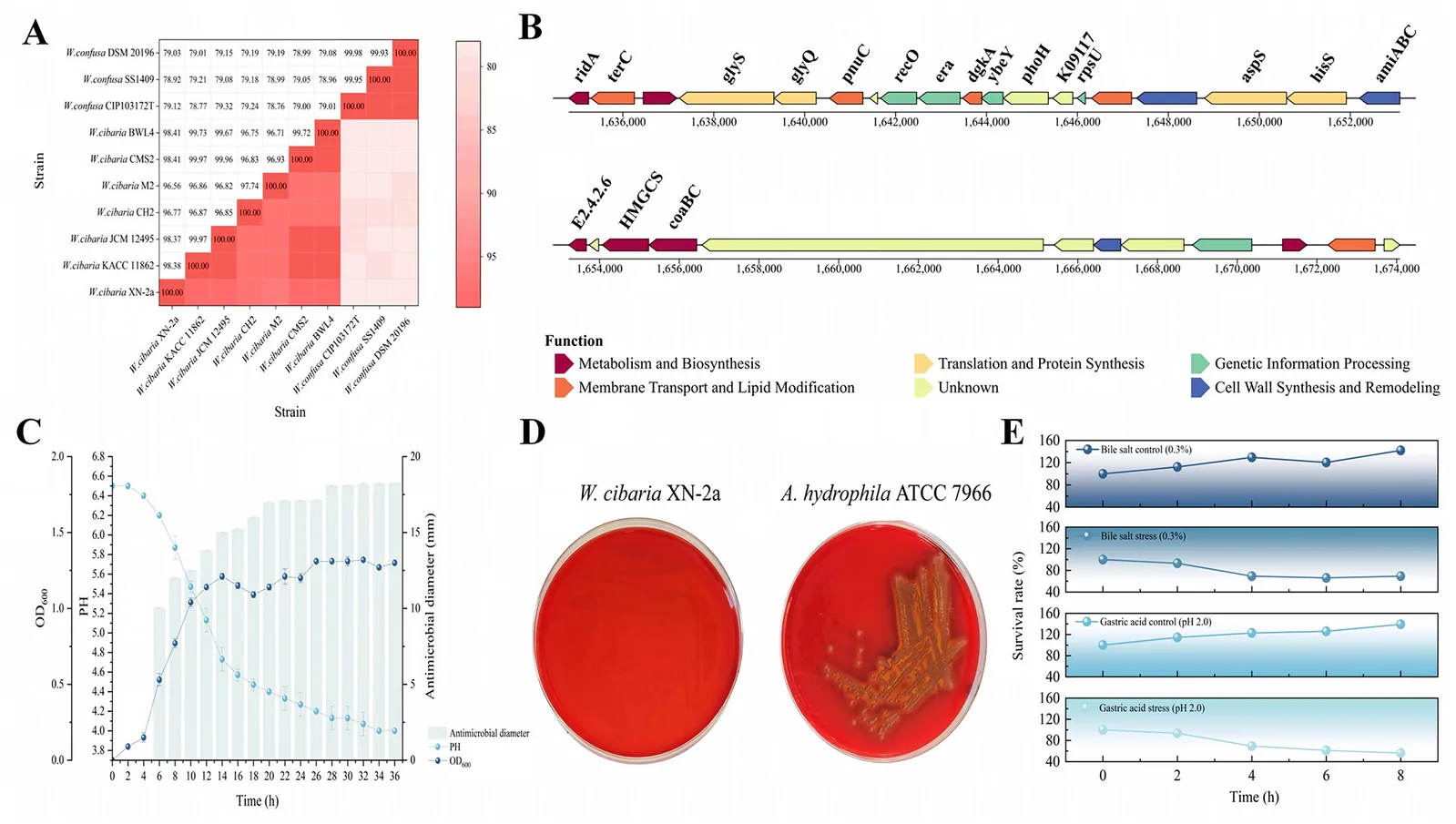
Genomic and pxhenotypic characterization of probiotic strain *W. cibaria* XN-2a. **(A)** Average nucleotide identity (ANI) heatmap comparing strain XN-2a with nine reference *Weissella* strains. Values above the diagonal indicate pairwise ANI (%), and the color gradient reflects similarity levels. The 95% species delineation threshold is indicated. **(B)** Genetic organization of the 41-kb type III polyketide synthase (T3PKS) biosynthetic gene cluster on the chromosome of XN-2a. Genes are color-coded by predicted function. **(C)** Growth kinetics (OD_600_, dark blue line), pH change (light blue line), and antimicrobial activity (blue shaded area, inhibition zone diameter in mm) of XN-2a against *A. hydrophila* ATCC 7966. Data are mean ± SD (*n* = 3). **(D)** Hemolytic activity on sheep blood agar. Left: XN-2a (no clear zone, γ-hemolysis); right: *A. hydrophila* (positive control, β-hemolysis). **(E)** Tolerance to simulated gastrointestinal conditions. Survival rates (%) of XN-2a after exposure to pH 2.0 (gastric acid) or 0.3% oxgall (bile salts) over 8 h, relative to control conditions (pH 6.5 or no bile salts). Data are mean ± SD (*n* = 3).

Growth kinetics showed a typical “S” curve: the strain entered logarithmic phase after 4 h, grew exponentially until 14 h, and then reached stationary phase. The medium pH decreased continuously from 6.5 to 4.0. Antimicrobial activity against *A. hydrophila* ATCC 7966 appeared at 6 h, reached 17.00 mm at 20 h, and peaked at 36 h (18.25 mm inhibition zone) (Figure 1C). No hemolytic activity was detected on sheep blood agar, suggesting the absence of direct hemolytic potential (Figure 1D). The strain exhibited good tolerance to simulated gastrointestinal conditions: after 8 h at pH 2.0, survival was 56.2%; after 8 h in 0.3% oxgall, survival was 69.2% (Figure 1E). Collectively, these results establish XN-2a as a safe, acid- and bile-tolerant, BLIS-producing probiotic candidate.

### 3.2 *In vitro* antibacterial mechanism of BLIS from *W. cibaria* XN-2a

As shown in Figure 2A, the BLIS exhibited moderate heat stability. After incubation at 40 °C and 60 °C for 1 h, the residual inhibition zones against *A. hydrophila* were 20.10–20.40 mm and 19.50–20.20 mm, respectively, comparable to the untreated control (20.19–20.60 mm). Even after heating at 100 °C for 1 h, the BLIS retained partial activity (9.70–10.50 mm).

**Figure 2:**
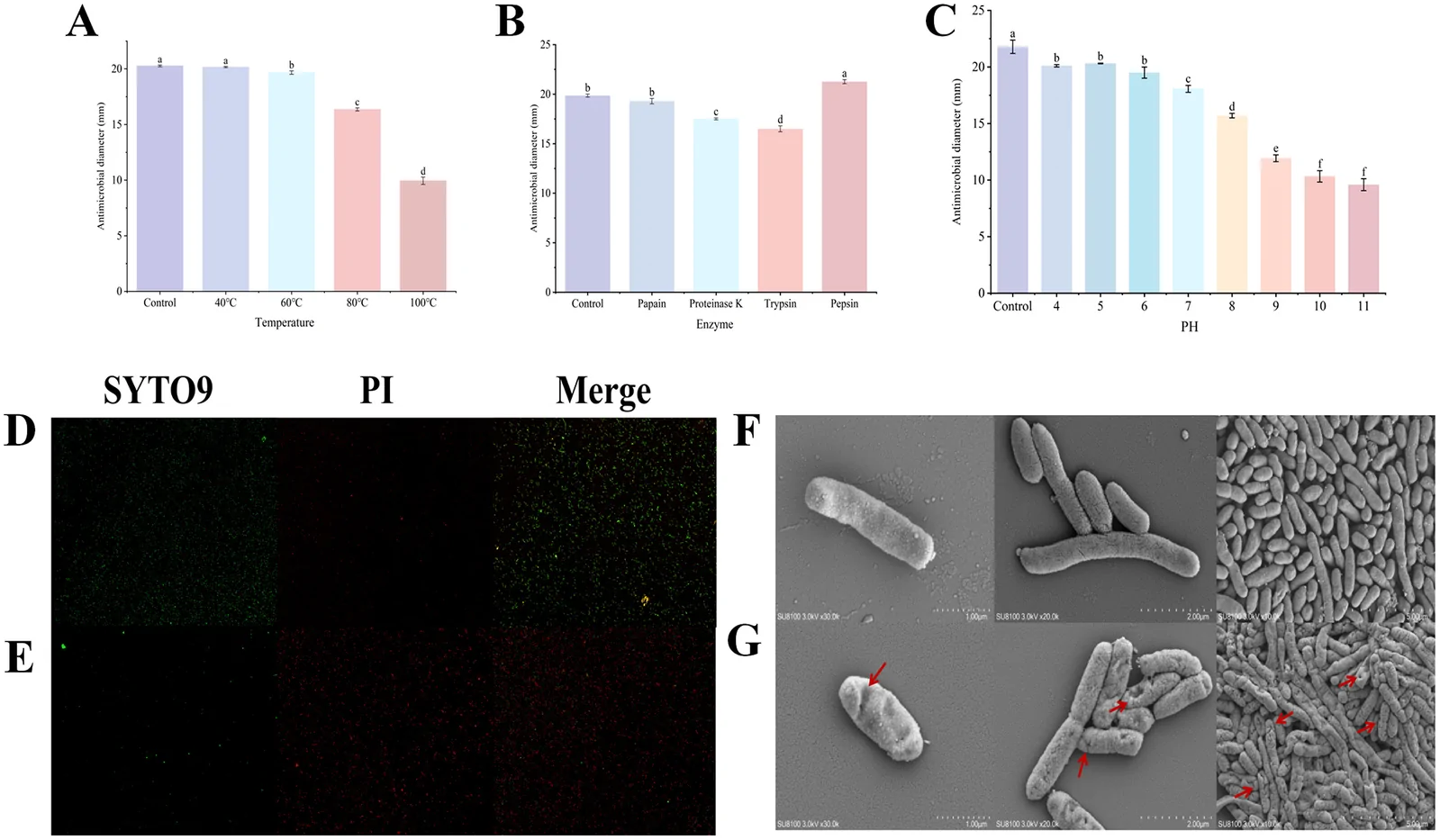
*In vitro* antibacterial mechanism of the BLIS from *W. cibaria* XN-2a against *A. hydrophila* ATCC 7966. **(A)** Thermal stability. Residual activity after 1 h incubation at 40–100 °C. **(B)** Protease sensitivity. Residual activity after treatment with proteinase K, trypsin, papain, or pepsin (1 mg/mL, 37 °C, 2 h). Different letters indicate significant differences (*p* ≤ 0.05). **(C)** Effect of pH on BLIS activity. Residual inhibition zone diameters after 2 h exposure to pH 4.0–11.0. **(D, E)** SYTO-9/PI live/dead staining. **(D)** Control group (green, intact membranes). **(E)** BLIS-treated group (red, membrane-damaged cells). Scale bar = 10 μm. **(F, G)** Scanning electron microscopy (SEM) images. **(F)** Untreated control cells showing smooth, intact surfaces. **(G)** BLIS-treated cells (2 h) exhibiting severe wrinkling, pore formation, and collapse. Scale bar = 1 μm.

The proteinaceous nature of the BLIS was confirmed by its sensitivity to proteolytic enzymes (Figure 2B). Treatment with proteinase K reduced the inhibition zone to 17.40–17.60 mm, while trypsin treatment caused a more pronounced decrease to 16.20–16.80 mm. Papain also significantly reduced activity (19.10–19.60 mm). In contrast, pepsin had no effect, with the BLIS retaining strong activity (21.10–21.50 mm) comparable to the control (19.70–20.00 mm). These results confirm that the antimicrobial substance is proteinaceous, thus classifying it as a BLIS.

Regarding pH sensitivity (Figure 2C), the BLIS was highly stable under acidic conditions (pH 4.0–6.0), producing inhibition zones of 19.01–20.34 mm. However, activity gradually declined when the pH exceeded 7.0, with inhibition zones of 15.50–15.90 mm at pH 8.0 and only 9.00–10.00 mm at pH 11.0, indicating that the BLIS is sensitive to alkaline environments.

The loss of membrane integrity was assessed using SYTO-9/PI double staining (Figures 2D, E). In the untreated control group, almost all cells emitted bright green fluorescence, indicating intact membranes and high viability (Figure 2D). After 2 h of BLIS exposure, a striking shift from green to red fluorescence was observed, reflecting PI influx into membrane-damaged cells (Figure 2E). This demonstrates that the BLIS severely impairs inner membrane permeability.

The effect of BLIS on bacterial membrane integrity was directly visualized by scanning electron microscopy (Figures 2F, G). Untreated *A. hydrophila* cells displayed a typical rod-shaped morphology with smooth, intact surfaces and clear outlines (Figure 2F). In contrast, after 2 h of BLIS treatment, the cells exhibited severe morphological alterations, including extensive wrinkling, collapse, surface roughening, and the formation of visible pores and hollow regions (Figure 2G). Some cells appeared partially lysed or completely distorted, providing direct evidence that the BLIS compromises membrane structural integrity.

Collectively, these findings establish that membrane disruption is the primary mechanism by which the BLIS of *W. cibaria* XN-2a exerts its bactericidal activity against *A. hydrophila*.

### 3.3 Growth performance, immune and antioxidant parameters

After the 30-day feeding trial, dietary supplementation with *W. cibaria* XN-2a significantly improved growth performance compared to the control group. As shown in Figure 3A, the experimental group exhibited a markedly higher WG (28.17% vs. 13.98%, *p* < 0.001) and a slightly but not significantly higher SR (96.67% vs. 93.33%, *p* > 0.05). In Figure 3B, the SGR was significantly increased in the probiotic-fed group (0.83%/day vs. 0.44%/day, *p* < 0.001), while the FCR was substantially reduced (1.88 ± 0.32 vs. 2.58 ± 0.27, *p* < 0.05), indicating superior feed efficiency. These results show that dietary *W. cibaria* XN-2a was associated with significantly higher WG, SGR, and lower FCR compared to the control group. Survival rates did not differ significantly between groups.

**Figure 3:**
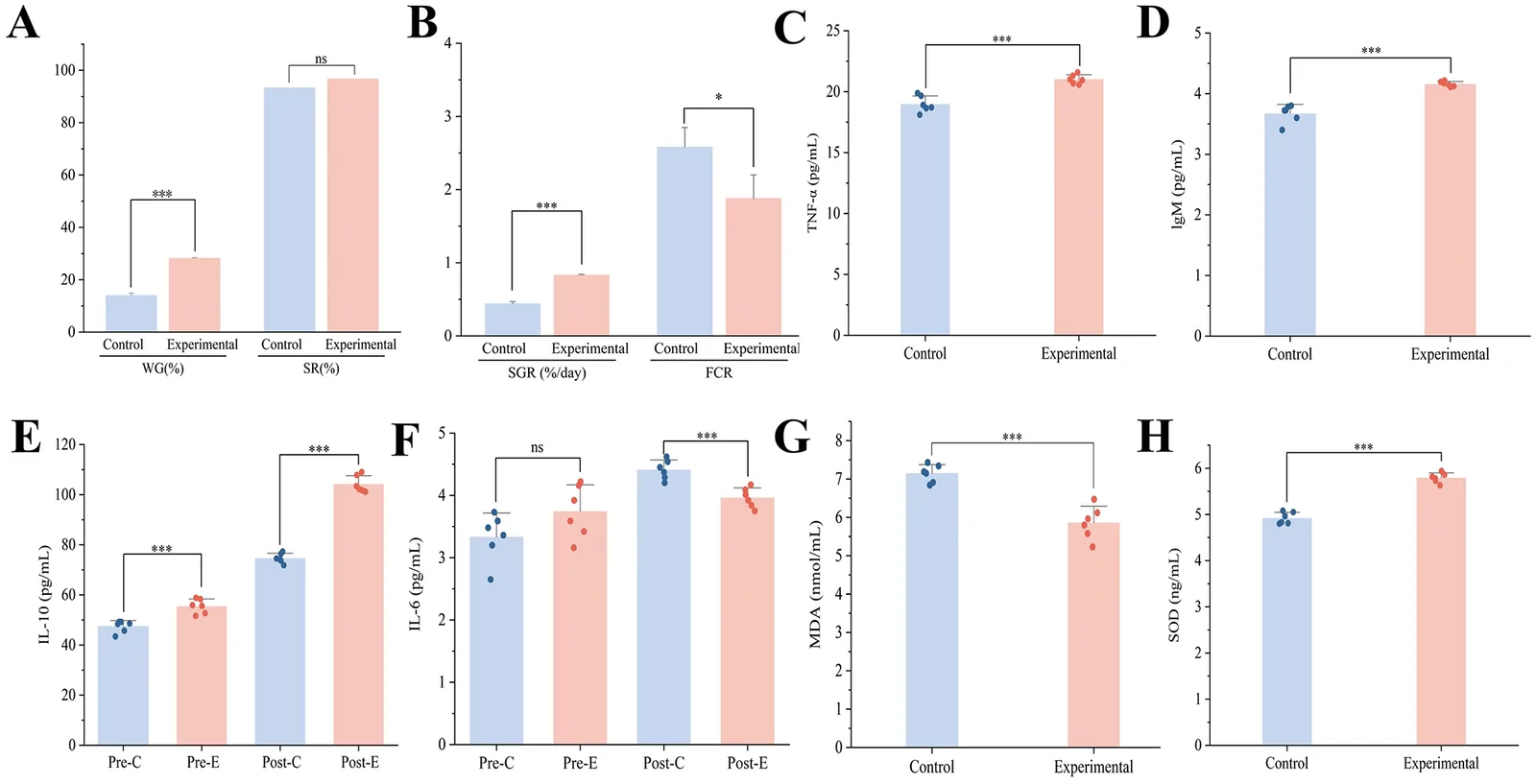
Growth performance, immune, and antioxidant parameters of crucian carp fed a control diet or a diet supplemented with *W. cibaria* XN-2a for 30 days. **(A)** Weight gain (WG, %) and survival rate (SR, %). **(B)** Specific growth rate (SGR, %/day) and feed conversion ratio (FCR). **(C–H)** Immune and antioxidant indices. **(C)** TNF-α, **(D)** IgM, **(E)** IL-10, **(F)** IL-6, **(G)** MDA, **(H)** SOD. For IL-6 and IL-10, values are shown before (Pre-C, Pre-E) and after (Post-C, Post-E) *A. hydrophila* challenge. For all other parameters, bars represent the basal levels without challenge. Experimental group (light red bars) vs. control group (light blue bars). Scattered dots indicate individual raw values. For IL-6 and IL-10 **(E, F)**, statistical significance was determined by two-way repeated measures ANOVA with Bonferroni *post-hoc* test (Table 3); for other panels, independent samples *t*-test was used. Data are mean ± SD (*n* = 3 replicate tanks per group). ^*^indicates *p* < 0.05, ^*^^*^^*^indicates *p* < 0.001, and ns, indicates not significant (*p* > 0.05).

Regarding humoral immunity and inflammatory markers, the probiotic-fed group exhibited significantly higher IgM concentration (4.12 ± 0.05 vs. 3.68 ± 0.19, *p* < 0.001), as shown in Figure 3D, reflecting strengthened systemic adaptive immunity. TNF-α levels were also moderately but significantly elevated in the experimental group (21.00 ± 0.71 vs. 19.00 ± 0.71, *p* < 0.001) (Figure 3C), which, together with the lower post-challenge IL-6 and higher IL-10, suggests an immune-priming effect rather than pathological inflammation.

The levels of inflammatory and anti-inflammatory cytokines were measured before and after *A. hydrophila* challenge. As summarized in Table 3, two-way repeated measures ANOVA for IL-6 revealed a significant main effect of Challenge (*F*(1.5) = 36.634, *p* = 0.002) and a significant Challenge × Group interaction (*F*(1.5) = 7.832, *p* = 0.038), while the main effect of Group was not significant (*F*(1,5) = 0.015, *p* = 0.906). Simple effect analysis further showed that IL-6 levels were comparable between the two groups before challenge (*p* = 0.103), but were significantly lower in the probiotic-fed group than in the control group after challenge (*p* < 0.001) (Figure 3F). For IL-10 (Figure 3E), two-way repeated measures ANOVA (Table 3) revealed significant main effects of Challenge (*F*(1,5) = 6641.799, *p* < 0.001) and Group (*F*(1,5) = 589.529, *p* < 0.001), as well as a significant Challenge × Group interaction (*F*(1,5) = 56.233, *p* < 0.001). Simple effect analysis demonstrated that the probiotic-fed group had significantly higher IL-10 levels than the control group both before and after challenge (*p* < 0.001 for both comparisons).

**Table 3:** Two-way repeated measures ANOVA results for IL-6 and IL-10 expression in crucian carp.

Indicator	Effect	*F*(1.5)	*P* value
IL-6	Challenge	36.634	0.002
	Group	0.015	0.906
	Challenge × group	7.832	0.038
IL-10	Challenge	6,641.799	< 0.001
	Group	589.529	< 0.001
	Challenge × group	56.233	< 0.001

*p* values were derived from two-way repeated measures ANOVA. df values are presented as (1, 5). *p* < 0.01 indicates highly significant; *p* < 0.05 indicates significant; *p* > 0.05 indicates not significant.

Oxidative stress markers are presented in Figures 3G, H. SOD activity was significantly higher in the probiotic group (5.80 ± 0.10 vs. 4.90 ± 0.10, *p* < 0.001) (Figure 3H), while MDA content was significantly lower (5.90 ± 0.22 nmol/mL vs. 7.15 ± 0.22 nmol/mL, *p* < 0.001) (Figure 3G), which is consistent with enhanced antioxidant capacity and reduced oxidative damage. These results show the probiotic's positive effects on growth and immune parameters.

### 3.4 Protective effects of *W. cibaria* XN-2a on intestinal integrity and digestive capacity

The protective effect of dietary *W. cibaria* XN-2a on intestinal health was evaluated by histopathological examination and measurement of digestive enzyme activity. As shown in Figures 4A–D, before challenge, both the control group (Figure 4A) and the experimental group (Figure 4B) exhibited intact intestinal morphology with well-aligned, slender mucosal folds, only scattered inflammatory cells in the mucosa, and a normal distribution of goblet cells, indicating a healthy baseline. After challenge, the control group (Figure 4C) developed severe intestinal damage, whereas the experimental group after challenge (Figure 4D) showed markedly alleviated injury. These observations indicate that fish in the experimental group showed less severe intestinal damage after challenge compared to the control group.

**Figure 4:**
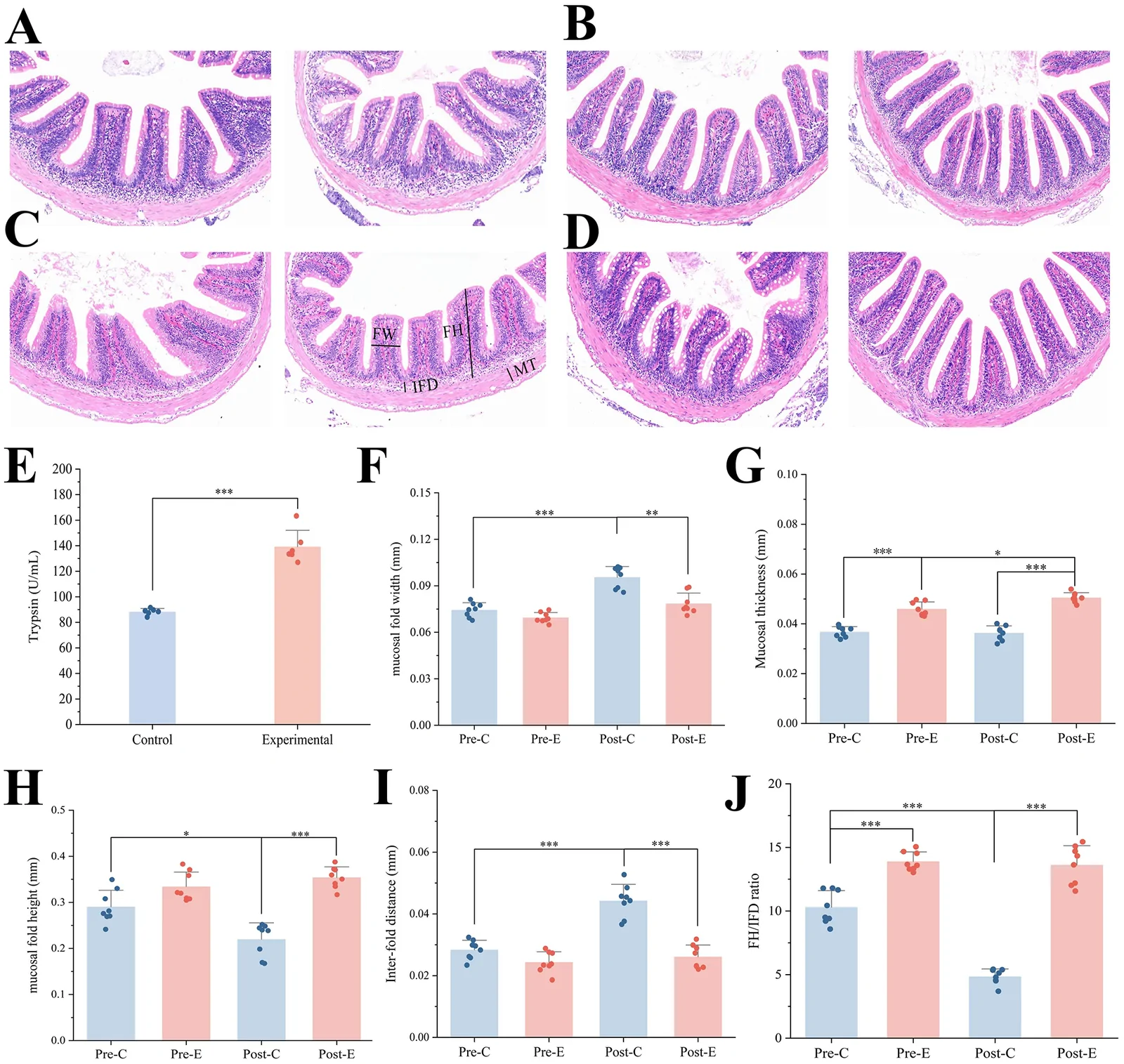
Intestinal morphology, digestive enzyme activity, and histopathology of crucian carp before and after *A. hydrophila* challenge. **(A–D)** Representative H&E-stained sections of distal intestine. **(A)** Control pre-challenge (Pre-C). **(B)** Experimental pre-challenge (Pre-E, fed XN-2a diet). **(C)** Control post-challenge (Post-C). **(D)** Experimental post-challenge (Post-E). Scale bar = 100 μm. **(E)** Trypsin activity. **(F)** mucosal fold width (FW). **(G)** Mucosal thickness (MT). **(H)** mucosal fold height (FH). **(I)** inter-fold distance (IFD). **(J)** mucosal fold height to inter-fold distance ratio (FH/IFD). For panels **(E–J)**, bars represent group means (*n* = 8 fish per group) with individual data points overlaid. For intestinal morphology parameters **(F–J)**, statistical significance was determined by two-way repeated measures ANOVA with Bonferroni *post-hoc* test (Table 4); for trypsin activity **(E)**, independent samples t-test was used. *p* < 0.001, *p* < 0.01, *p* < 0.05; ns, not significant. ^*^indicates *p* < 0.05, ^*^^*^indicates *p* < 0.01, and ^*^^*^^*^indicates *p* < 0.001.

Digestive function, as indicated by trypsin activity, was significantly enhanced by probiotic supplementation. As shown in Figure 4E, the experimental group exhibited markedly higher trypsin activity (138.00 ± 12.41 U/mL) compared to the control group (88.00 ± 3.08 U/mL, *p* < 0.001), indicating improved protein digestion capacity.

Quantitative analysis of intestinal architecture (Figures 4F–J) further confirmed the protective effects. Two-way repeated measures ANOVA (Table 4) revealed significant main effects of Challenge and Group, as well as significant Challenge × Group interactions for all intestinal morphology parameters (FW: *F*(1.7) = 7.013, *p* = 0.033; MT: *F*(1.7) = 15.550, *p* = 0.006; FH: *F*(1.7) = 18.614, *p* = 0.004; IFD: *F*(1.7) = 31.986, *p* < 0.001; FH/IFD: *F*(1.7) = 41.937, *p* < 0.001), indicating that the effect of probiotic supplementation on intestinal morphology was dependent on the challenge status. Simple effect analysis further revealed that pathogen challenge induced significant alterations in the control group. Compared with pre-challenge values (Pre-C), the control group after challenge (Post-C) showed a marked decrease in FH (Figure 4H) and in the FH/IFD (Figure 4J) (*p* < 0.001 for both), together with a significant increase in IFD (Figure 4I) and FW (Figure 4F) (*p* < 0.001 for both). MT (Figure 4G) remained unchanged in the control group after challenge (*p* = 1.000). In contrast, the experimental group maintained most morphometric parameters at levels comparable to the healthy pre-challenge state, with no significant differences between Pre-E and Post-E for FH (*p* = 1.000), IFD (*p* = 1.000), FH/IFD (*p* = 1.000), and FW (*p* = 0.056), while MT showed a significant increase after challenge (*p* = 0.034). Simple effect analysis also showed that before challenge, the experimental group already exhibited significantly higher MT (*p* < 0.001) and FH/IFD (*p* < 0.001) than the control group, while FW showed no significant difference (*p* = 0.570) and FH showed no significant difference (*p* = 0.156). After challenge, all indices in the experimental group were significantly superior to those in the control group: MT, FH, and FH/IFD were higher (*p* < 0.001, *p* < 0.001, and *p* < 0.001, respectively), while IFD and FW were lower (*p* < 0.001 and *p* = 0.002, respectively). These results indicate that fish in the probiotic-fed group showed better preservation of mucosal fold structure, increased muscular thickness, lower crypt hyperplasia, reduced mucosal fold edema, and a healthier FH/IFD ratio compared to the control group.

**Table 4:** Two-way repeated measures ANOVA results for intestinal morphology parameters (FW, MT, FH, IFD, and FH/IFD) in crucian carp.

Indicator	Effect	*F*(1.7)	*P* value
FW	Challenge	150.886	< 0.001
	Group	37.549	< 0.001
	Challenge × group	7.013	0.033
MT	Challenge	24.934	0.002
	Group	97.156	< 0.001
	Challenge × group	15.550	0.006
FH	Challenge	8.658	0.022
	Group	44.733	< 0.001
	Challenge × group	18.614	0.004
IFD	Challenge	45.236	< 0.001
	Group	104.682	< 0.001
	Challenge × group	31.986	< 0.001
FH/IFD	Challenge	66.567	< 0.001
	Group	896.169	< 0.001
	Challenge × group	41.937	< 0.001

*p* values were derived from two-way repeated measures ANOVA. df values are presented as (1, 7). *p* < 0.05 indicates significant; *p* < 0.01 indicates highly significant; *p* < 0.001 indicates extremely significant.

### 3.5 Protection against *A. hydrophila* challenge

To evaluate the protective efficacy of dietary *W. cibari*a XN-2a, fish from both groups were challenged with *A. hydrophila* ATCC 7966 via intraperitoneal injection of 0.1 mL bacterial suspension at a concentration of 1.5 × 10^8^ CFU/mL (equivalent to 1.5 × 10^7^ CFU per fish). Mortality was recorded daily for 7 days. The sham-injected control group (PBS) exhibited no mortality throughout the 7-day observation period (data not shown), confirming that the observed mortality was specifically attributable to *A. hydrophila* infection. The Kaplan-Meier survival curves for both groups are shown in Figure 5. The control group exhibited rapid mortality, reaching 100% cumulative mortality by day 7. In contrast, the probiotic-fed group showed significantly lower mortality, with a final cumulative mortality of 50%. The log-rank (Mantel-Cox) test confirmed a significant difference in survival between the two groups (χ^2^ = 17.4, *p* < 0.001). The relative level of protection (RLP) was calculated as 50%. These results indicate that dietary supplementation with *W. cibaria* XN-2a provided moderate but significant protection against *A. hydrophila* infection in crucian carp.

**Figure 5:**
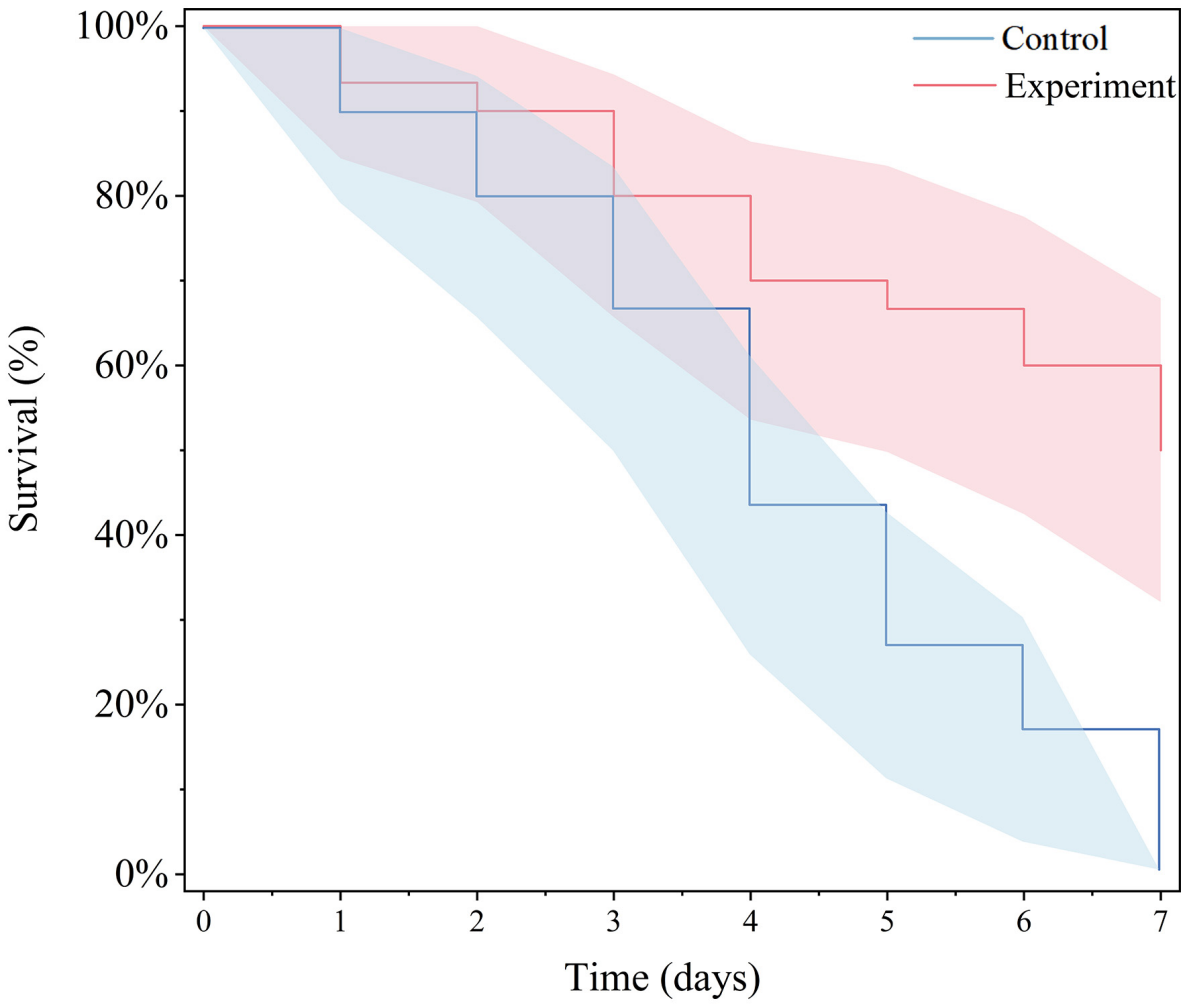
Kaplan-Meier survival curves of fish challenged with *A. hydrophila* over a 7-day observation period. The blue solid line represents the control group, and the red solid line represents the experimental group (probiotic-fed group). Shaded areas indicate the 95% confidence intervals (95% CI) for survival rates. The x-axis represents time (days) post-challenge, and the y-axis represents the survival rate (%). Survival data were analyzed using the Kaplan-Meier method, and differences between groups were evaluated by the log-rank (Mantel-Cox) test. The results indicate that the survival rate of the experimental group was significantly higher than that of the control group (χ^2^ = 17.40, *p* < 0.001).

### 3.6 *W. cibaria* XN-2a shapes the gut microbiota, resistome and virulence profile

Alpha diversity measured by the Shannon index showed no significant difference between the XS and XD groups (*p* > 0.05, Figure 6A). Beta diversity analysis by PCoA based on Bray-Curtis distances revealed a clear separation between the two groups (Figure 6B). PC1 explained 40.03% and PC2 explained 25.07% of the variation. ANOSIM confirmed a significant difference (*p* < 0.0001). These results indicate that while overall alpha diversity remained unchanged, the overall community structure was reshaped by probiotic supplementation.

**Figure 6:**
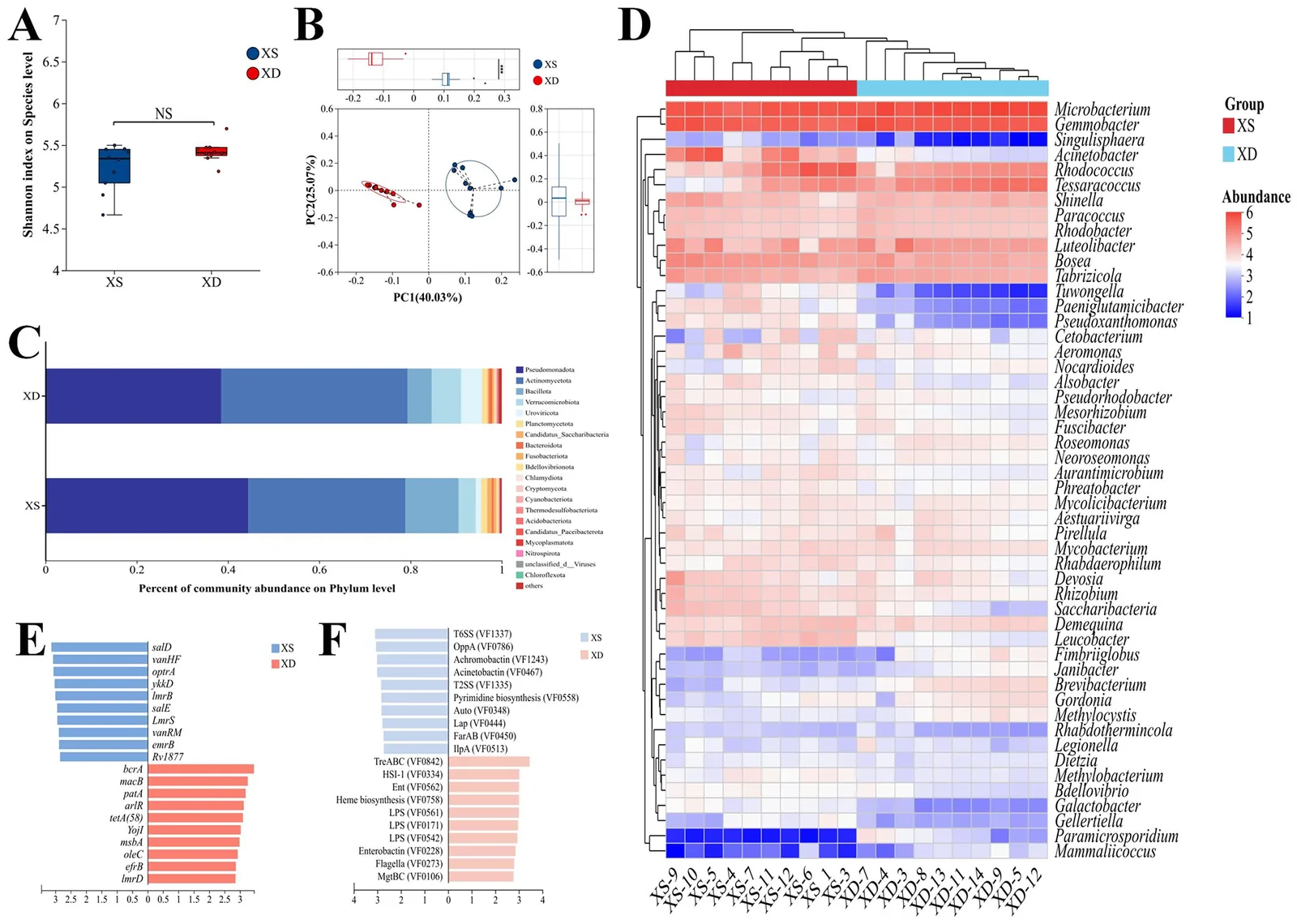
Gut microbiota structure, resistome, and virulence potential. **(A)** Shannon index (alpha diversity) at the species level. XS, probiotic-supplemented group; XD, control group. NS, no significant difference (*p* > 0.05). **(B)** Principal coordinate analysis (PCoA) of beta diversity based on Bray-Curtis distances at the genus level, with 95% confidence ellipses. ANOSIM, *p* < 0.0001. **(C)** Phylum-level relative abundance (stacked bars). **(D)** Heatmap of the top 30 genera with hierarchical clustering. Color gradient from blue (low) to red (high) indicates normalized abundance. **(E)** LEfSe analysis of differentially abundant antibiotic resistance genes (ARGs, CARD database, LDA > 2, *p* < 0.05). Blue bars, enriched in XS; red bars, enriched in XD. *vanHF, vanH* gene in the *vanF* cluster; LmrS, *Staphylococcus aureus* LmrS; *vanRM, vanR* gene in the *vanM* cluster. **(F)** LEfSe analysis of differentially abundant virulence factors (VFs, VFDB database, LDA > 2, *p* < 0.05). Blue bars, enriched in XS; red bars, enriched in XD. OppA, oligopeptide-binding protein OppA; TreABC, trehalose-recycling ABC transporter.

At the phylum level (Figure 6C), the dominant phyla in both groups were Pseudomonadota, Actinomycetota, and Bacillota (>80% combined). The XS group had higher relative abundances of Pseudomonadota, Bacillota, and Saccharibacteria, while the XD group was enriched in Actinomycetota, Verrucomicrobiota, Uroviricota, Bacteroidota, and Fusobacteriota. The genus-level heatmap (Figure 6D) separated XS from XD samples into two clusters. XS was characterized by higher abundance of *Singulisphaera, Tuwongella, Paeniglutamicibacter, Pseudoxanthomonas, Devosia, Rhizobium, Saccharibacteria, Demequina, Leucobacter, Dietzia, Methylobacterium, Bdellovibrio, Galactobacter*, and *Gellertiella*. XD was enriched in *Fimbriiglobus, Janibacter, Brevibacterium, Gordonia, Methylocystis, Paramicrosporidium*, and *Mammaliicoccus*. Thus, a marked shift in taxonomic composition occurred at both phylum and genus levels.

LEfSe analysis of ARGs (Figure 6E) showed that XS was enriched in *salD, vanHF, optrA, ykkD, lmrB, salE, LmrS, vanRM, emrB* and *Rv1877*. XD was enriched in *bcrA, macB, patA, arlR, tetA*(*58*), *YojI, msbA, oleC, efrB* and *lmrD*. For virulence factors (Figure 6F), XS was enriched in T6SS (VF1337), OppA (VF0786), achromobactin (VF1243), acinetobactin (VF0467), T2SS (VF1335), pyrimidine biosynthesis (VF0558), Auto (VF0348), Lap (VF0444), FarAB (VF0450), and IlpA (VF0513). XD was enriched in trehalose ABC transporter (VF0842), HSI-1 (VF0334), Ent (VF0562), heme biosynthesis (VF0758), three LPS entries (VF0561, VF0171, VF0542), enterobactin (VF0228), flagella (VF0273), and MgtBC (VF0106). LEfSe analysis identified different sets of ARGs and VFs enriched in the XS vs. XD groups (Figures 6E, F). The functional implications of these differences are considered in the Discussion.

### 3.7 Metagenomic differences in KEGG pathways, butanoate metabolism, and CAZy gene abundances

KEGG pathway analysis revealed a clear functional separation between the XS and XD groups (PCoA, Figure 7A; ANOSIM, *p* < 0.001). LEfSe analysis at Level 3 (Figure 7B) showed that XS was enriched in pathways related to signaling and energy metabolism (e.g., Two-component system, Ribosome, Quorum sensing, Fatty acid metabolism), whereas XD was enriched in secondary metabolism and biosynthesis pathways (e.g., Biosynthesis of secondary metabolites, Streptomycin biosynthesis, Galactose metabolism).

**Figure 7:**
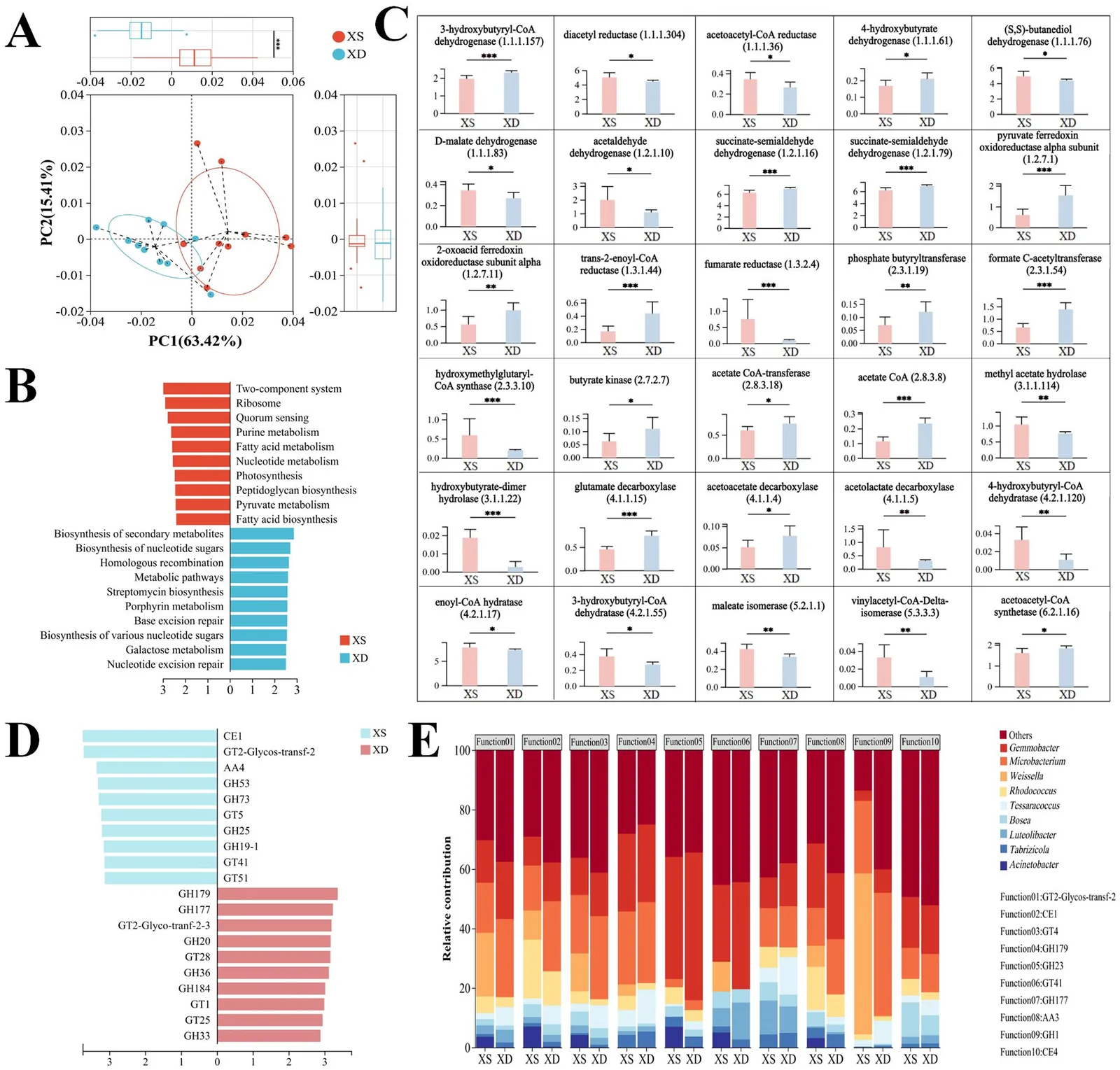
Functional reprogramming of the gut microbiota by *W. cibaria* XN-2a supplementation. **(A)** PCoA of KEGG pathway profiles (Level 2) based on Bray-Curtis distances. Ellipses: 95% confidence. ANOSIM, *p* < 0.001. **(B)** LEfSe analysis of differentially abundant KEGG pathways (Level 3, LDA > 2, *p* < 0.05). Red bars, enriched in XS; blue bars, enriched in XD. **(C)** Butanoate metabolism pathway (ko00650). Bar chart presents key enzyme abundances (mean relative abundance ± SD) with significance levels from Wilcoxon test (*p* < 0.05, *p* < 0.01, *p* < 0.001). **(D)** LEfSe analysis of differentially abundant CAZy families (LDA > 2, *p* < 0.05). Blue bars, enriched in XS; red bars, enriched in XD. **(E)** Species contribution to ten selected CAZy families (stacked bars). Each bar represents the relative contribution (%) of different genera. ^*^indicates *p* < 0.05, ^*^^*^indicates *p* < 0.01, and ^*^^*^^*^indicates *p* < 0.001.

In the butyrate metabolism pathway (ko00650), XS showed higher abundances of genes encoding butyrate-synthetic enzymes, including acetoacetyl-CoA reductase (1.1.1.36, *p* < 0.05), enoyl-CoA hydratase (4.2.1.17, *p* < 0.001), acetolactate decarboxylase (4.1.1.5, *p* < 0.001), and hydroxymethylglutaryl-CoA synthase (2.3.3.10, *p* < 0.001). In contrast, XD was enriched in genes encoding butyrate kinase (2.7.2.7, *p* < 0.05) and phosphate butyryltransferase (2.3.1.19, *p* < 0.01) (Figure 7C)

CAZy analysis (Figure 7D) showed that XS was enriched in GH25, GH73, AA4, and CE1, whereas XD was enriched in GH179 and GH177. Species contribution analysis (Figure 7E) revealed that Weissella was a major contributor to several CAZy families enriched in XS, but contributed to none in XD.

In summary, the XS and XD groups differed in KEGG pathways, butanoate metabolism, and CAZy profiles. These metagenomic differences reflect genetic potential rather than functional activity, and their biological significance requires further investigation.

## 4 Discussion

In this study, we investigated the probiotic potential of *W. cibaria* XN-2a in crucian carp through a combination of *in vitro* characterization, *in vivo* feeding trial, and multi-omics analyses. The results demonstrate that dietary supplementation with this BLIS-producing strain improves growth performance, intestinal morphology, immune function, and disease resistance, accompanied by directed remodeling of the gut microbiota and its metabolic potential. We first examined the strain's genetic features to understand the molecular basis of its probiotic-related phenotypes.

The genome of *W. cibaria* XN-2a contains a complete type III polyketide synthase (T3PKS) gene cluster spanning approximately 39 kb (positions 1,635,000–1,674,000 bp; Figure 1B). T3PKS gene clusters have also been identified in other *Weissella* species, including *W. confusa* (Tenea, 2026), suggesting that this biosynthetic capacity may be conserved within the genus. Beyond the core T3PKS biosynthetic genes (e.g., *HMGCS and coaBC* for extender unit supply), this cluster encodes a remarkable array of auxiliary genes that collectively may contribute to the strain's probiotic functionality. These include genes involved in metabolism and biosynthesis (*ridA*, EC 2.4.2.6), membrane transport and lipid modification (*terC, pnuC, dgkA*), translation and protein synthesis (*glyS, glyQ, aspS, hisS, rpsU*), genetic information processing (*recO, era, ybeY*), and cell wall synthesis/remodeling (*amiABC*).While the presence of these genes is consistent with the observed acid and bile tolerance, their functional contribution requires confirmation through gene expression analysis or targeted mutagenesis (Gokhale et al., 2007; Bruna et al., 2024).

*In vitro* experiments confirmed that the strain produces a BLIS that directly lyses *A. hydrophila* by disrupting membrane integrity, as evidenced by scanning electron microscopy (severe wrinkling, pore formation, and collapse) and SYTO-9/PI staining (increased membrane permeability; Figures 2D–G). T3PKS products are often phenolic lipids with broad-spectrum antimicrobial and antioxidant activities, in contrast to the classical peptide bacteriocins (e.g., plantaricin, pediocin) (Tenea, 2026; Gokhale et al., 2007). Some polyketides, such as ε-poly-L-lysine, even exhibit bacteriostatic and biodegradable properties that have led to their use as food preservatives (Chaichana et al., 2025). In this context, it is plausible that the BLIS of XN-2a contributes to both direct bactericidal and antioxidant functions, which is consistent with the reduced oxidative stress (lower MDA, higher SOD) observed in the probiotic-fed group.

Moreover, the strain displayed considerable tolerance to simulated gastric acid (pH 2.0, 56 % survival at 8 h) and bile salts (0.3 % oxgall, 69 % survival at 8 h; Figure 1E). These values are comparable or superior to those of several commercial probiotic strains, suggesting its potential for gastrointestinal survival. The presence of *terC* (heavy metal tolerance), *pnuC* (NAD^+^ salvage), and *recO* (DNA repair) in the T3PKS cluster may contribute to this stress resilience. The absence of hemolytic activity further supports the safety of XN-2a, classifying it as a safe and beneficial strain for food and feed applications (Anglenius et al., 2023; Chaichana et al., 2025). Collectively, the T3PKS gene cluster and its auxiliary components provide the genetic and metabolic foundation for effective intestinal colonization, pathogen antagonism, and subsequent microbiota remodeling. These properties, together with the improved growth performance, enhanced immune parameters, and better intestinal morphology documented in the probiotic-fed crucian carp, support *W. cibaria* XN-2a as a promising probiotic candidate for sustainable aquaculture. Nevertheless, it must be emphasized that while the presence of these genes is consistent with the observed acid and bile tolerance and BLIS activity, their functional contribution *in vivo* requires confirmation through gene expression analysis or targeted mutagenesis.

Although alpha diversity (Shannon index) remained unchanged, beta diversity (PCoA) showed a clear separation between the XS and XD groups, indicating that dietary *W. cibaria* XN-2a induced a selective structural shift in the gut microbiota rather than random drift. Notably, the predatory genus *Bdellovibrio*, which preys on Gram-negative pathogens, was significantly enriched in the XS group. Adding *Bdellovibrio* to aquaculture water reduces culturable bacteria, improves survival after *A. hydrophila* challenge, and decreases Enterobacterales abundance (Zhang et al., 2023). Since *Bdellovibrio* was absent from the control diet and not deliberately added, its enrichment in XS suggests that *W. cibaria* XN-2a creates a favorable niche for this complementary biocontrol agent, thereby enhancing host resistance. This observation highlights how a BLIS-producing probiotic can indirectly promote the establishment of a second, complementary biocontrol agent, a phenomenon that may be harnessed to design multi-layered prophylactic strategies in aquaculture. Importantly, the preservation of overall alpha diversity together with this beneficial compositional shift strongly supports the safety and efficacy of this BLIS-producing strain as a feed additive.

The virulence factor (VF) profiles provided strong evidence for a shift from a pathogenic to a commensal configuration. The XD group was heavily enriched in LPS (lipopolysaccharide), flagella, enterobactin and heme-acquisition systems—a typical virulence-associated gene set of Gram-negative opportunistic pathogens such as *A. hydrophila*. LPS is a potent stressor in fish that compromises immune function and induces oxidative stress, ultimately leading to growth retardation and muscle deterioration (Zhang et al., 2025). Flagella and enterobactin promote tissue adhesion and iron acquisition. In contrast, the XS group was enriched in the T6SS and T2SS, alongside alternative siderophores (achromobactin, acinetobactin). T6SS is a contact-dependent nanomachine that injects toxic effectors into competing bacteria, mediating bacterial antagonism and helping to establish a stable niche (Hachani et al., 2016). In beneficial or commensal bacteria, T6SS often targets other microorganisms rather than the host, thus contributing to the competitive exclusion of pathogens. T2SS, widespread in bacteria, can be involved in scavenging-enzyme secretion and surface-protein assembly rather than delivering host-targeted toxins (Xiong et al., 2025). These observations are consistent with a scenario in which probiotic supplementation is associated with a functional shift of the microbial community away from host-targeted virulence factors and toward inter-bacterial competition mechanisms, potentially lowering the overall pathogenic potential.

The resistome analysis further supports this interpretation. The overall ARG class composition remained similar between the two groups, a favorable safety indicator. The ARGs enriched in XS—such as *optrA*, LmrS and *lmrB*—are commonly carried by lactic acid bacteria as part of their core genome and often encode BLIS-export pumps rather than clinically acquired resistance determinants. Indeed, the multidrug resistance transporter LmrB in *Lactococcus lactis* functions in both BLIS secretion and immunity (Gajic et al., 2003). By analogy, the enrichment of these genes in XS likely reflects the active secretion of bacteriocins by *W. cibaria* XN-2a, supporting its probiotic function. In contrast, the XD group harbored ARGs such as *bcrA, macB, patA, arlR, tetA*(*58*), *YojI, msbA, oleC, efrB* and *lmrD* (Figure 6E), frequently detected in opportunistic fish pathogens, indicating a higher baseline resistance load. Together, these ecological changes indicate that *W. cibaria* XN-2a administration enriches BLIS-related resistance genes intrinsic to lactic acid bacteria while curbing pathogen-associated ARGs, thereby enhancing host protection without increasing the overall resistome burden. This safety-oriented probiotic strategy offers a sustainable alternative to antibiotic growth promoters in aquaculture.

Building on the compositional changes described above, we further analyzed differences in metagenomic functional potential. A notable metagenomic difference between the two groups was the butanoate metabolism pathway (ko00650). The XS group showed higher abundances of genes encoding butyrate-synthetic enzymes (e.g., enoyl-CoA hydratase, acetolactate decarboxylase), whereas the XD group was enriched in genes associated with butyrate diversion or degradation (Figure 7C). These metagenomic observations suggest that this pattern is consistent with an increased genetic potential for butyrate synthesis in the XS microbiota. Butyrate is well documented for its anti-inflammatory, barrier-enhancing, and growth-promoting effects in aquatic animals (Zhu et al., 2021). Dietary supplementation with sodium butyrate or Clostridium butyricum has been shown to improve intestinal morphology, antioxidant capacity, and disease resistance in crucian carp, turbot, rainbow trout, and Pacific white shrimp, as well as to enhance growth and immune responses in tilapia, common carp, yellow catfish, and large yellow croaker (Tran et al., 2023; Liu et al., 2019; Ke et al., 2022; Qiu et al., 2026). These effects are directionally consistent with butyrate's known functions, although direct causation remains to be established.

Similarly, CAZy analysis revealed that the XS group was enriched in GH25, GH73 (lysozyme-like enzymes), AA4 (auxiliary redox enzymes), and CE1 (carbohydrate esterases), whereas the XD group was enriched in families associated with complex polysaccharide degradation (GH179, GH177) (Figure 7D). GH25 and GH73 hydrolyse peptidoglycan and exert lytic activity against Gram-positive bacteria (Yang et al., 2025; Kmezik et al., 2021); AA4 enzymes generate hydrogen peroxide and participate in oxidative metabolism; and CE1 releases fermentable sugars from hemicelluloses (Mai-Gisondi et al., 2017). The concurrent enrichment of these families in XS is directionally consistent with enhanced genetic potential for antibacterial and antioxidant functions, particularly in the context of a BLIS-producing strain.

Nevertheless, it must be emphasized that both the butanoate pathway and CAZy data reflect gene abundances rather than actual enzymatic activities or metabolite concentrations. Direct quantification of butyrate (e.g., by GC-MS) and functional validation of CAZy families through enzymatic assays, targeted metabolomics, or *in vitro* culture experiments are required before any mechanistic conclusions can be drawn. Therefore, these metagenomic observations should be considered hypothesis-generating rather than definitive mechanistic links to the phenotypic improvements observed in this study.

The microbial and metabolic reprogramming observed in the gut of *W. cibaria* XN-2a-fed fish translated into clear, quantifiable improvements in intestinal architecture, immune status and growth performance, as summarized in Figures 3A–H, 4F–J. These host phenotypes are consistent with the probiotic's efficacy and may be linked to the antimicrobial and immunomodulatory activities of the BLIS.

Improvements in intestinal morphology (Figures 4F–J) were accompanied by increased trypsin activity (Figure 4E), indicating better protein digestion and amino acid availability. Two-way repeated measures ANOVA (Table 4) confirmed significant Challenge × Group interactions for all intestinal morphology parameters, indicating that the beneficial effects of probiotic supplementation on intestinal structure were modulated by the challenge status. Butyrate (a potential end-product of the butanoate pathway that was enzymatically enriched in the XS group) is known to promote GLP-2 secretion and subsequent crypt cell proliferation and villus growth (Abdel-Latif et al., 2020). A healthy villus-height-to-crypt-depth ratio is a well-established indicator of absorptive surface area and barrier integrity (Tran et al., 2023).

The immune profile (Figures 3C–H) showed a primed pattern. Higher baseline IL-6 before challenge (not statistically significant) suggested immune priming, while lower post-challenge IL-6 and elevated IL-10 indicated a controlled, non-excessive inflammatory response that limits tissue damage without compromising pathogen clearance (Jiang Y. H. et al., 2024). Elevated IgM was consistent with enhanced humoral immunity, and the combination of higher SOD and lower MDA indicated reduced oxidative stress. Such a low-inflammation, high-antioxidant status favors energy allocation to growth over immune-mediated catabolism (Amoah et al., 2019).

Improved growth performance (Figures 3A, B) was consistent with enhanced digestive activity (higher trypsin), expanded absorptive area, and reduced metabolic cost of inflammation. Previous studies have linked improved growth with probiotic modulation of the GH/IGF-1 axis (Mahfuj et al., 2026). Together, these host phenotypes are consistent with the microbial and metabolic changes described above. It should be noted that although we did not independently analyze the commercial diet, prior studies using the same feed support that relative comparisons remain valid when both groups receive the same batch (Qi et al., 2025; Jiang K. M. et al., 2024).

Based on the correlative multi-omics and phenotypic data presented above, we propose a working hypothesis that integrates the main findings. Dietary *W. cibaria* XN-2a may reach and transiently persist in the distal intestine and produces a BLIS (supported by *in vitro* activity), which may suppress certain pathogenic or competing bacteria. This suppression could create a niche for butyrate-producing taxa, consistent with the metagenomic enrichment of butyrate-synthesis-associated enzymes (Figure 7C). If higher butyrate levels are indeed generated (a point requiring direct quantification), this metabolite could contribute to the improved intestinal barrier (higher FH/FW, lower inter-fold distance) and the reduced post-challenge IL-6 observed in the XS group. In parallel, the enrichment of GH25, GH73 and AA4 in the CAZy profile of XS may further support antibacterial and anti-oxidative defenses. Nevertheless, it must be emphasized that this putative sequence has not been directly proven in the present study. Alternative explanations are equally plausible, and causal relationships remain to be tested using butyrate quantification, BLIS-gene knockout mutants, and gnotobiotic models.

Several limitations should be noted: butyrate was not measured; BLIS production was demonstrated *in vitro* but not confirmed *in vivo*; the T3PKS cluster expression in the fish gut remains to be investigated; microbiota differences are correlational and the lack of controls (heat-killed bacteria or a non-BLIS-producing mutant) prevents attributing effects solely to viable *W. cibaria* or its BLIS; immune and histological parameters were measured only at limited time points; and the trial was conducted under controlled laboratory conditions, requiring field studies for real-world validation. Therefore, the integrated model should be viewed as hypothesis-generating, not definitive.

Despite these limitations, the main findings of this study are clear. In conclusion, dietary supplementation with *W. cibaria* XN-2a was associated with significantly improved growth performance, feed efficiency, immune function and intestinal health of crucian carp, and provided moderate (50%) protection against *A. hydrophila* challenge. The strain's production of a heat- and acid-stable BLIS with membrane-disrupting activity represents a key functional attribute that likely contributes to these beneficial effects. The probiotic did not increase the overall antibiotic resistance burden and was associated with a selective reduction of pathogen-associated virulence factors. Metagenomic analysis revealed differences in the butanoate metabolism pathway and CAZy profiles between the control and probiotic-fed groups, which suggest an increased potential for butyrate synthesis and antibacterial/antioxidant activities. These metagenomic observations provide a foundation for future hypothesis-driven studies. *W. cibaria* XN-2a represents a promising probiotic candidate for sustainable aquaculture, and its antimicrobial protein-producing capability warrants further investigation for potential applications in feed formulations.

## Data Availability

The 16S rRNA gene sequence is available in NCBI GenBank (accession: PX884722), while the whole-genome and metagenomic sequencing data are available in the NCBI SRA (accession numbers listed in Table 2).
